# Regulation of Granulocyte and Macrophage Populations of Murine Bone Marrow Cells by G-CSF and CD137 Protein

**DOI:** 10.1371/journal.pone.0015565

**Published:** 2010-12-13

**Authors:** Dongsheng Jiang, Herbert Schwarz

**Affiliations:** 1 Department of Physiology, and Immunology Programme, Yong Loo Lin School of Medicine, Singapore, Singapore; 2 NUS Graduate School for Integrative Sciences and Engineering, National University of Singapore, Singapore, Singapore; Ohio State University, United States of America

## Abstract

**Background:**

Granulocytes and monocytes/macrophages differentiate from common myeloid progenitor cells. Granulocyte colony-stimulating factor (G-CSF) and CD137 (4-1BB, TNFRSF9) are growth and differentiation factors that induce granulocyte and macrophage survival and differentiation, respectively. This study describes the influence of G-CSF and recombinant CD137-Fc protein on myelopoiesis.

**Methodology/Principal Findings:**

Both, G-CSF and CD137 protein support proliferation and survival of murine bone marrow cells. G-CSF enhances granulocyte numbers while CD137 protein enhances macrophage numbers. Both growth factors together give rise to more cells than each factor alone. Titration of G-CSF and CD137 protein dose-dependently changes the granulocyte/macrophage ratio in bone marrow cells. Both factors individually induce proliferation of hematopoietic progenitor cells (lin^-^, c-kit^+^) and differentiation to granulocytes and macrophages, respectively. The combination of G-CSF and CD137 protein further increases proliferation, and results in a higher number of macrophages than CD137 protein alone, and a lower number of granulocytes than G-CSF alone demonstrating that CD137 protein-induced monocytic differentiation is dominant over G-CSF-induced granulocytic differentiation. CD137 protein induces monocytic differentiation even in early hematopoietic progenitor cells, the common myeloid progenitors and the granulocyte macrophage progenitors.

**Conclusions/Significance:**

This study confirms earlier data on the regulation of myelopoiesis by CD137 receptor - ligand interaction, and extends them by demonstrating the restriction of this growth promoting influence to the monocytic lineage.

## Introduction

Granulocytes are essential cells of the innate immune system. As neutrophil and eosinophil granulocytes they form the first defense line against bacteria and multicellular parasites, respectively. Through release of their cytotoxic and inflammatory mediators granulocytes participate in the elimination of pathogens, recruitment of additional immune cells and perpetuation of the inflammatory reaction [1]. The activity of granulocytes is partly regulated via their life span which is short under normal conditions. Neutrophils, which constitute about 95% of all granulocytes, have a half life of a just few hours in circulation. At sites of inflammation proinflammatory cytokines such as G-CSF, granulocyte macrophage colony-stimulating factor (GM-CSF), tumor necrosis factor (TNF) and interferon (IFN)-γ extend the life span of granulocytes by preventing apoptosis [2], [3]. Numbers of granulocytes can also be increased by enhancing the proliferation rate of hematopoietic progenitor cells and their differentiation rate to granulocytes. G-CSF is the single most important factor for inducing the generation of new granulocytes from bone marrow. G-CSF is also used to treat neutropenia induced by cancer chemo or radiation therapy [4].

CD137, a member of the TNF receptor family, can be expressed by several types of hematopoietic cells, and is involved in the regulation of multiple and diverse types of immune responses [5], [6]. CD137 ligand is expressed as a transmembrane molecule on the surface of antigen presenting cells and it too delivers signals into APC [7], [8]. Signaling of CD137 ligand induced by recombinant CD137 protein or anti-CD137 ligand antibodies enhances B cell proliferation, and activation, survival and migration of monocytes [9]–[16]. CD137 ligand agonists also induce differentiation of peripheral human monocytes to mature dendritic cells (DCs) [17], [18] as well as DC maturation [19]–[21].

CD137 and its ligand not only influence mature immune cells but also play a role in hematopoiesis. Expression of CD137 and its ligand have been found in the bone marrow [22]–[24], but different studies report different conclusions of the functions of the CD137 receptor/ligand system in the bone marrow and in hematopoiesis. While some studies report an inhibitory effect of CD137 ligand signaling on myelopoiesis [22], [24] others find that the CD137 receptor/ligand system induces proliferation of hematopoietic progenitor cells, colony formation of colony-forming unit (CFU) granulocyte/macrophage (CFU-GM) and CFU macrophage (CFU-M), and myelopoiesis resulting the generation of monocytes and macrophages [23], [25].

The other myeloid cell type besides monocytes/macrophages that originate from CFU-GM are granulocytes. Based on the enhancing effects of CD137 on other myeloid cells and its role in regulating survival and apoptosis of mature granulocytes [26] we aimed to determine how CD137 might influence the generation of granulocytes.

We find that treatment of total murine bone marrow cells with recombinant CD137 protein enhances the percentage of myeloid cells except that of granulocytes. G-CSF and CD137 protein work together in stimulating cell proliferation and survival. The underlying mechanisms are (1) a cell type-specific promotion of cell survival by G-CSF and CD137 protein, and (2) the induction of monocytic rather than granulocytic differentiation of early hematopoietic progenitor cells by CD137 protein.

## Materials and Methods

### Mice

Female BALB/c mice between 8 and 16 weeks of age were used as a source of bone marrow cells. Animals were specific pathogen free, and kept with free access to food and water in the animal care facility at the National University of Singapore under the institutional guidelines for usage of experimental animals under protocol 078-06.

### Isolation of bone marrow CD11b^+^, Ly6G^+^ cells, lin^-^, c-kit^+^ cells, CMP and GMP

The femur bones of BALB/c mice were dissected and the bone marrow was flushed out with phosphate-buffered saline (PBS), 2 mM EDTA by using a 10 ml syringe and 27G needle. Total bone marrow cells were passed through 30 µm filter (Miltenyi Biotec, Germany), washed with PBS, 2 mM EDTA and resuspended in RPMI1640 (Sigma), 10% fetal bovine serum (FBS).

The CD11b^+^, Ly6G^+^ cells were isolated by immunomagnetic separation (MACS) of CD11b^+^ cells and followed by fluorescent activated cell sorting (FACS) of Ly6G^+^ cells. Briefly, the fresh bone marrow cells were incubated with anti-CD11b microbeads (Miltenyi) for 20 min. The cell suspension was passed through the LS column (Miltenyi) in strong magnetic field. The CD11b^+^ cells were retained in the column. After three washes, the column was removed from magnetic filed and CD11b^+^ cells were flushed out. The cells were stained with FITC-Ly6G and PE-CD11b Abs and the CD11b^+^, Ly6G^+^ cells were sorted by FACS on MoFlo XDP cell sorter (Beckman Coulter, CA, USA).

The lin^-^, c-kit^+^ cells were enriched from bone marrow cells by two steps MACS, with depletion of mature cells with lineage markers followed by selection of CD117^+^ (c-kit) cells, as described previously [23]. The enriched cells were stained with Pacific blue-conjugated anti-mouse CD34, PE-conjugated anti-mouse CD117, PerCP-Cy5.5-conjugated anti-mouse CD16/32, and biotinylated lineage Abs followed by FITC-conjugated Extravidin, and then were subjected to FACS sorting on MoFlo XDP cell sorter (Beckman Coulter). The lin^-^, c-kit^+^, CD34^+^, CD16/32^low^ population was collected as common myeloid progenitors (CMP) and the lin^-^, c-kit^+^, CD34^+^, CD16/32^high^ population was collected as granulocyte-macrophage progenitors (GMP).

Bone marrow cells, CD11b^+^, Ly6G^+^ cells, lin^-^, c-ckit^+^ cells, CMPs and GMPs were cultured in RPMI1640 (Sigma) supplemented with 10% fetal bovine serum (FBS), 2 mM L-glutamine, 100 U/ml penicillin and 100 µg/ml streptomycin.

### Recombinant proteins and cytokines

Recombinant human CD137-Fc protein was purified from supernatants of stable transfected CHO cells by protein G sepharose, as described previously [27]. The endotoxin concentration in the CD137-Fc protein is 55 IU/mg. Human IgG1 Fc protein was purchased from Accurate Chemical and Scientific Corporation (Westbury, NY, USA). Recombinant mouse G-CSF was purchased from Peprotech (Rocky Hill, NJ, USA).

### Antibodies and flow cytometry

FITC-conjugated anti-mouse Ly-6G (clone 1A8), PE-conjugated anti-mouse Gr-1 (clone RB6-8C5), CD11b (clone M1/70), CD14 (clone Sa2-8), F4/80 (clone BM8), CD137 ligand (clone TKS-1), APC-conjugated anti-mouse CD115 (clone AFS98), Pacific blue-conjugated anti-mouse CD34 (clone RAM34), PerCP-Cy5.5-conjugated anti-mouse CD16/32 (clone 93) antibodies and respective isotype controls (rat IgG2a, rat IgG2b), and PE-conjugated hamster anti-mouse CD137 (clone 17B5) and its isotype control (Golden Syrian hamster IgG) were purchased from eBioscience (San Diego, CA). PE-conjugated anti-mouse CD117 (clone 3C1) and cocktail of biotinylated anti-mouse lineage markers were obtained from Miltenyi (Germany). FITC-conjugated Extravidin was purchased from Sigma (MO, USA). FITC-conjugated anti-mouse MPO (clone 8F4) and its isotype mouse IgG1 were obtained from Hycult biotech (Netherlands).

For cell surface staining, the Fc receptors were first blocked by FcR blocking reagent (Miltenyi, Germany) and incubated with Ag-specific Abs for 30 min at 4°C. For intracellular staining, the manufacture instructions of the Fixation/Permeabilization kit from BD Biosciences (NJ, USA) were followed. The nonspecific staining was controlled by isotype-matched antibodies.

Flow cytometry was performed either on a FACSCalibur (BD Biosciences) with CellQuest data acquisition and analysis software, or on a Cyan flow cytometer (Beckman Coulter) with Summit software.

### Cell count

#### Manual cell count

Cells were harvested after incubation with 10 mM EDTA for 10 min. Cells were centrifuged and resuspended in PBS. The numbers of viable cells were assessed by 0.4% Trypan blue (Sigma) staining and counted by using a Neubauer haemocytometer.

#### Cell count by flow cytometry

Cell samples were stained with 0.5 µg/ml 7-AAD for 15 min at room temperature in the dark and were resuspended in 450 µl PBS. 50 µl of Sphero AccuCount Blank Particles (about 50,000 beads per 50 µl), (ACBP-100-10, Spherotech, IL, USA) were added to the suspension and mixed well. Samples were analyzed by flow cytometry with CyAn™ ADP Analyzer (Beckman Coulter). 7-AAD negative cells were gated as live cells. The population of beads and target cells were gated separately on the forward/side scatter plot based on different sizes. The number of cells in each samples was calculated by the formula: Number of cells in sample  =  (Number events of test sample x Number of beads)/Number of events of beads.

### Proliferation assay

Cell proliferation was determined by ^3^H-thymidine incorporation. Cells were pulsed with 0.5 µCi of ^3^H-thymidine (PerkinElmer, Boston, MA) for the last 24 h of the culture period. The cells were then harvested onto a Packard Unifilter Plate using a MicroMate 196 Cell Harvester and counted using a TopCount Microplate Scintillation Counter (Packard Instruments, Meriden, CT).

### Esterase stain

2–5×10^6^ cells were smeared onto glass slides. Esterase stain was performed successively with the α-Naphyl Acetate Esterase kit (Sigma) for non-specific esterase and the Naphthol AS-D Chloroacetate Esterase kit (Sigma) for specific esterase following the manufacturer's instructions.

### Apoptosis assay

Purified bone marrow CD11b^+^, Ly6G^+^ cells were cultured in 10 µg/ml CD137-Fc precoated wells for 4, 16 and 24 h. Cells were harvested and labeled with Annexin V-PE and 7-AAD (BD Bioscience) and analyzed by flow cytometry measuring, with the compensation for FITC-Ly6G.

### Microscopy and photography

Cell morphology and esterase stain were documented by using a Zeiss Axiovert 40 inverted microscope (Zeiss, Germany) and Canon PowerShot G6 digital camera.

### Statistics

Unless otherwise stated, two tailed unpaired Student's t-tests were used to determine statistical significance.

## Results

### Stimulation of bone marrow cells with CD137 protein leads to an increase in the percentage of myeloid cell except of granulocytes

**Figure 1:**
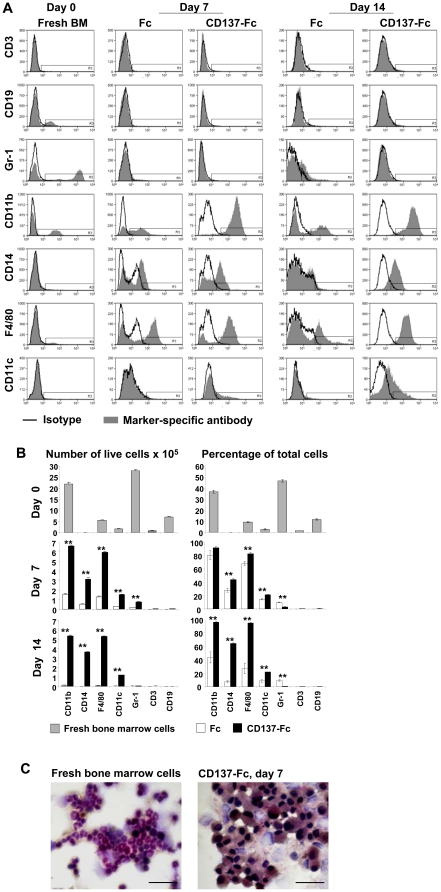
Recombinant CD137 protein enhances the percentage of monocytic cells but not that of granulocytes. (A) Bone marrow cells were analyzed immediately after isolation (day 0) for cell surface marker expression by flow cytometry, or 6×10^6^ bone marrow cells were cultured in T25 flasks coated with 1 ml of 10 µg/ml of Fc or CD137-Fc protein and analyzed on days 7 and 14. Black line: Isotype. Gray, filled curve: Marker-specific antibody. (B) Depicted are absolute numbers of live cells (left column) and percentages (right column) of data in (A) for indicated subpopulations at days 0, 7 and 14. (C) Fresh murine bone marrow cells, or cells which were cultured on plates coated with 10 µg/ml CD137-Fc protein for 7 days, smeared on glass slides, fixed with citrate-acetone-formaldehyde solution, and stained for non-specific esterase (black, macrophages) and for specific esterase (purple, granulocytes). Slides were counterstained with hematoxylin and mounted with cover slips. Magnification: ×630. Scale bar: 25 µm. These data are representative of three independent experiments with similar results.

Among freshly isolated bone marrow cells the most prominent subpopulations were Gr-1^bright^ granulocytes (46.7%), and CD11b^+^ myeloid cells (36.8%). However, most of the myeloid cells seemed to be immature since CD14 and F4/80, markers for monocytes and macrophages, were present only on 0.1% and 9.2% of the cells, respectively. CD11c, a marker for DCs was found only on 2.6% of the cells. 1.2% and 11.6% of the cells were T and B cells, respectively, as determined via CD3 and CD19 expression (Figure 1A,B).

For crosslinking of CD137 ligand we used a CD137-Fc fusion protein consisting of the extracellular domain of CD137 fused to the constant domain of human IgG1 (Fc) which was immobilized to the culture dishes. A Fc protein was used as a negative control, and mock coating with PBS, the solvent for the Fc and CD137-Fc proteins, was used as a medium control. At day 7, T and B cells had virtually disappeared (0.4% and 0.6%, respectively), indicating that lymphocyte survival and/or differentiation is not supported under these conditions. The percentage of granulocytes had decreased tremendously and was significantly lower in the CD137 condition (3.2% vs 9.9%) implying an inhibitory effect of CD137 protein on granulocyte development. Among the CD137 protein-treated cells, the percentages of cells positive for CD11b, CD14, F4/80 had increased to 91.4%, 43.9% and 82.2%, respectively. Among the Fc protein-treated cells, the percentages of cells positive for CD11b, CD14, F4/80 had also increased (79.8%, 27.7% and 67.7%, respectively) compared to fresh bone marrow cells, but to a lesser degree compared to CD137-Fc-treated cells (Figure 1A,B).

At day 14, granulocytes among the Fc protein-treated cells remained at 9.6%, whereas they were not detectable (0.7%) among the CD137 protein-treated cells (Figure 1A,B). Among the CD137 protein-treated cells the percentages of CD11b^+^, CD14^+^ and F4/80^+^ population had further increased to 95.8%, 64.2% and 94.6%, respectively. The percentage of DCs as detected by CD11c staining had increased from 2.6% to 21.8%. In contrast, among the Fc protein-treated cells the CD11b^+^, CD14^+^, F4/80^+^ populations were declined substantially. The Fc control protein was not able to support DC survival or differentiation (Figure 1A,B). Overall cell survival rate for Fc- and CD137-Fc-treated bone marrow cells were 3.2% and 12.0%, respectively, for day 7, and 0.4% and 9.3%, respectively, for day 14 (Figure 1B).

The CD137 protein-induced bias towards promoting survival of monocytic bone marrow cells could also be verified by esterase staining. Naphthol AS-D chloroacetate, a substrate for specific esterase, stains cells of the granulocytic lineage purple, and these cells were predominant in fresh bone marrow cells (Figure 1C). Also clearly visible in fresh bone marrow cells are the ring-shaped nuclei, typical for murine neutrophils. After a 7-day exposure to immobilized CD137 protein, most cells stained black with α-naphthyl acetate, a substrate for non-specific esterase demonstrating the shift towards the monocytic lineage. This is supported by the concomitant increase in cell size and the kidney-shaped nuclei which are typical of monocytes/macrophages.

### CD137 protein does not induce apoptosis of bone marrow granulocytes

**Figure 2:**
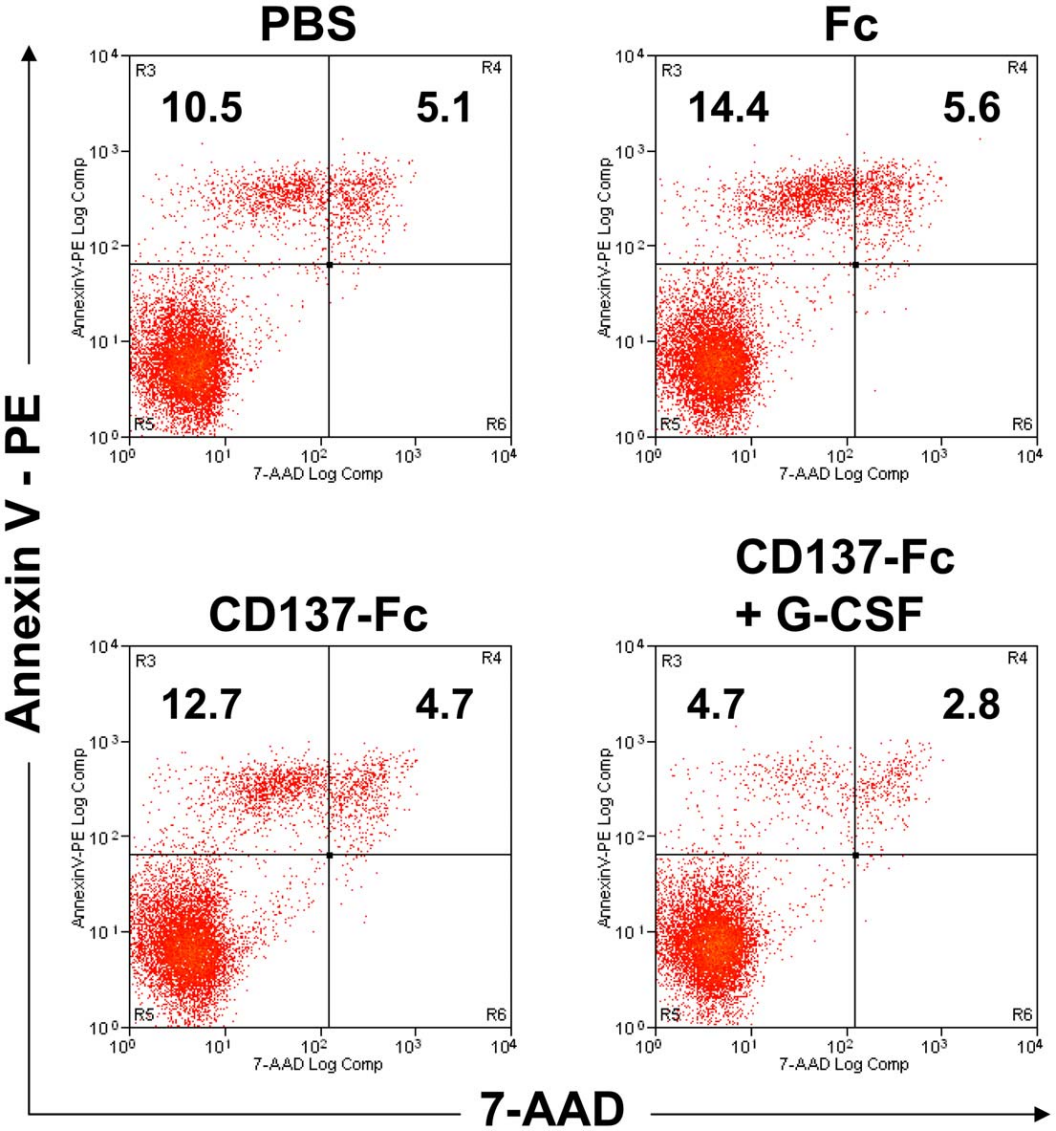
CD137 protein does not induce apoptosis of granulocytes. CD11b^+^ cells were enriched from bone marrow by MACS, and labeled with CD11b-PE and Ly6G-FITC Ab. 3×10^5^ FACS-sorted CD11b^+^, Ly6G^+^ cells at a density of 6×10^5^ cells/ml were cultured on plates coated with 10 µg/ml Fc or CD137-Fc protein for 24 h. Cells were stained with Annexin V-PE and 7-AAD and analyzed by flow cytometry with compensation of FITC channel. PBS: Medium control. This figure is a representative of three independent experiments.

Since a possible explanation for the decrease in the percentage of granulocytes could be CD137 protein-induced granulocyte apoptosis, we tested the effects of CD137 protein on granulocyte survival and death. CD137 protein induced neither cell death in granulocytes among bone marrow cells (not shown) nor in purified granulocytes over a 24 h period (Figure 2). However, addition of G-CSF reduced the rate of granulocyte apoptosis, and the percentage of early apoptotic cells (Annexin V^+^, 7-AAD^−^) dropped from 12.7 to 4.7%, and that for late apoptotic cells (Annexin V^+^, 7-AAD^+^) from 4.7 to 2.8% (Figure 2). The differences in apoptosis rates between the PBS, Fc and CD137-Fc conditions were not statistically significant among three independent experiments.

### G-CSF and CD137 protein cooperatively induce survival and proliferation of bone marrow cells

Since CD137 protein supports monocyte but not granulocyte survival and/or differentiation we investigated the interaction of CD137 protein with G-CSF, the classical granulocyte growth and differentiation factor, in regulating bone marrow cell survival, proliferation and differentiation.

**Figure 3:**
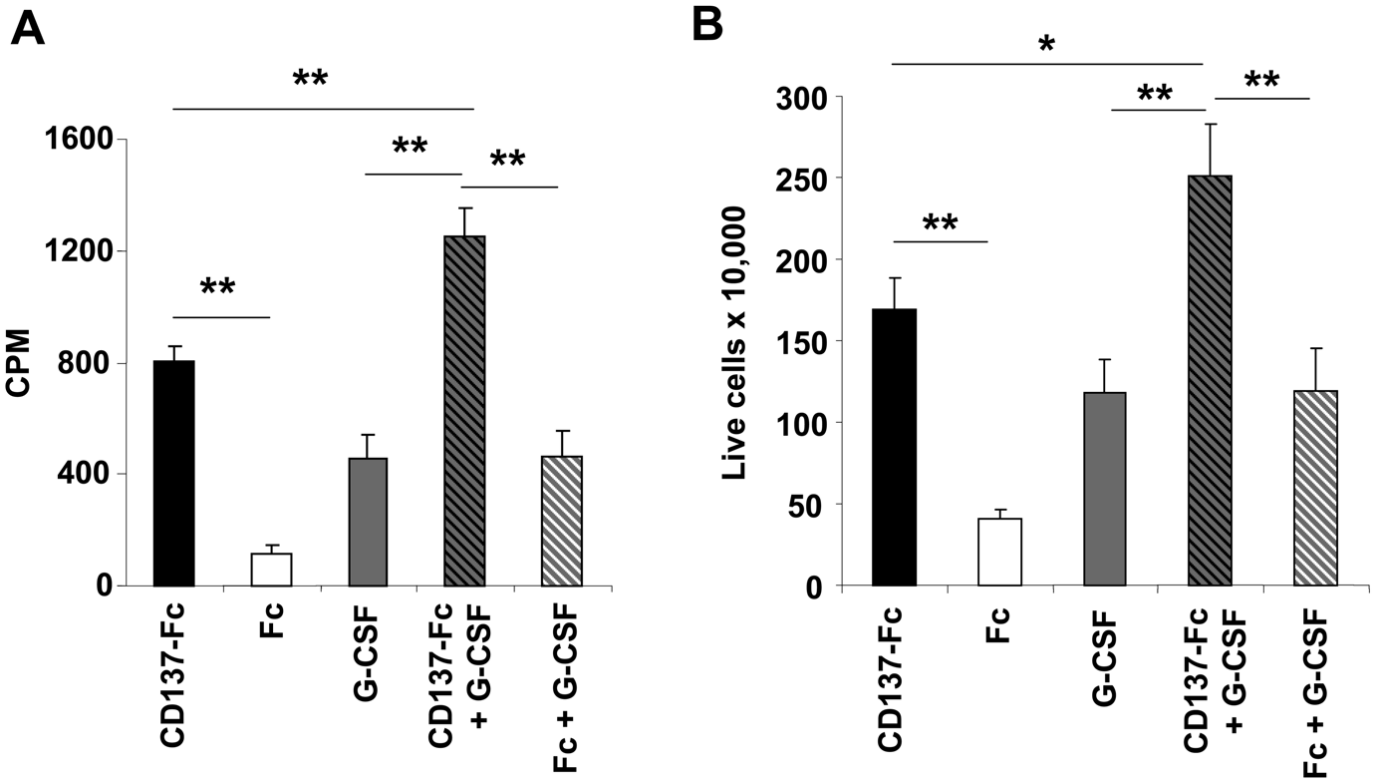
G-CSF and CD137 protein induce proliferation and survival of bone marrow cells. Murine bone marrow cells at a density of 10^6^ cells/ml were cultured for 7 days with respective treatment. 10 µg/ml of Fc or CD137-Fc protein were pre-coated on the plates. (A) Proliferation assay: 1 ng/ml of G-CSF was added daily to indicated wells. Cells (10^5^/condition) were labeled for the last 24 h with 0.5 µCi ^3^H-thymidine, and the rate of proliferation was determined with a scintillation counter (Packard, Meriden, CT). Depicted are means ± standard deviations of triplicate measurements. (B) Cell count: 10 ng/ml of G-CSF was added daily to indicated wells. Cells (2×10^6^/condition) were harvested on day 7, and cell count was determined by trypan blue staining of 4 representative microscopic fields. Depicted are means ± standard errors of seven independent experiments. * p<0.05; ** p<0.01.

Bone marrow cells were treated with one dose of 10 µg/ml of immobilized CD137-Fc protein or daily doses of 1 ng/ml G-CSF, or both. Cells treated with Fc protein or Fc + G-CSF were used as controls. Both CD137 protein and G-CSF increased cell proliferation (Figure 3A) and cell numbers (Figure 3B), with comparable potencies. The combination of CD137 protein and G-CSF was more potent than either factor alone (Figure 3A,B).

### CD137 protein and G-CSF enhance bone marrow macrophage and granulocyte numbers, respectively

**Figure 4:**
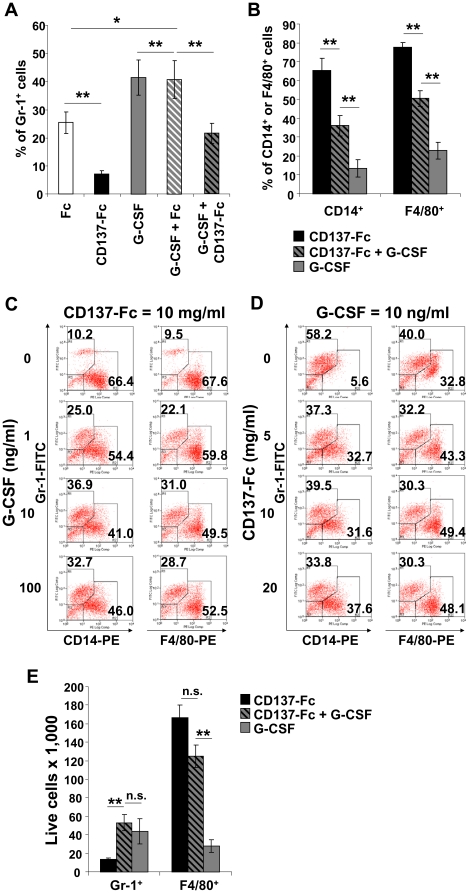
Effect of G-CSF and CD137 protein on granulocyte and macrophages numbers. 2×10^6^ murine bone marrow cells at a density of 10^6^ cells/ml were cultured for 7 days on plates that had been coated with 10 µg/ml of Fc or CD137-Fc protein. Where indicated, G-CSF was added daily at 10 ng/ml or other stated concentrations. The percentages of Gr-1^+^ cells (A) and CD14^+^, F4/80^+^ cells (B), as well as absolute cell numbers (E), determined by flow cytometry on day 7, are depicted as means ± standard errors of 7 independent experiments. Dose responses of the combination of G-CSF and CD137 protein: Cells were treated with a fixed concentration of CD137-Fc at 10 µg/ml and varying the concentration of G-CSF at 0, 1, 10, or 100 ng/ml (C); or a fixed concentration of G-CSF at 10 ng/ml and varying the concentration of CD137-Fc at 0, 5, 10, or 20 µg/ml (D), and flow cytometry was performed for Gr-1, CD14 and F4/80 on day 7. These data are representative of two experiments with similar results. Statistical significance was determined by two tailed, paired Student's t-test. * p<0.05; ** p<0.01; n.s. not significant.

Data in Figure 1 implied that CD137 protein reduces the percentage of granulocytes. This could indeed be verified by treating bone marrow cells for 7 days with CD137-Fc protein or G-CSF or both, and analyzing cell composition by flow cytometry via Gr-1 and CD14 and F/4/80 expression, markers for granulocytes and macrophages, respectively. Bone marrow cells treated with CD137 protein contained a significantly lower percentage of Gr-1^+^ cells than the Fc control condition (7.2±1.2% vs 25.4±3.9%), (Figure 4A). In bone marrow cells treated with G-CSF 41.5±6.2% of the cells were Gr-1^+^. The presence of CD137 protein however reduced the G-CSF-mediated increase in granulocyte percentage to 21.5±3.6%. The Fc control protein had no influence on the percentage of Gr-1^+^ cells (40.7±6.6%), (Figure 4A). Reciprocally, G-CSF reduced the CD137 protein-mediated increase in the macrophage percentage. Of CD137-Fc-treated bone marrow cells 65.4±5.4% and 77.9±2.4% expressed CD14 and F4/80, respectively, and these number dropped to 36.1±5.3% and 50.6±4.2%, respectively, when G-CSF was added (Figure 4B).

The effects of G-CSF and CD137 protein on the composition of the cell population are dose-dependent. Bone marrow cells were grown for 7 days on plates coated with a fixed concentration (10 µg/ml) of CD137-Fc or Fc protein, and G-CSF was added daily at 0, 1, 10 or 100 ng/ml. In the absence of G-CSF two thirds of the cells expressed CD14 and/or F4/80 and 10% expressed Gr-1. With increasing concentrations of G-CSF the percentage of granulocytes increased and the percentage macrophages decreased (Figure 4C). However, G-CSF at 100 ng/ml did not induce more granulocytes than at 10 ng/ml, indicating a saturation point between these two concentrations. In a parallel experiment G-CSF was kept constant at 10 ng/ml, and the coating concentrations of CD137-Fc and Fc proteins were varied from 0, 5, 10 and 20 µg/ml. In the absence of CD137 protein 58.2% expressed Gr-1 and 5.8% expressed CD14 (Figure 4D). Increasing concentrations of CD137 protein reduced the percentage of granulocytes and increased correspondingly the percentage of macrophages. The pattern was similar for F4/80 (Figure 4D).

The dose-dependent shift in the granulocyte/macrophage ratio by the titration of G-CSF and CD137 protein was predominantly due to a cell-type-specific survival effect that increased the total number of granulocytes and macrophages, respectively. Similar to the relative composition of cells, G-CSF treatment resulted in a high absolute number of Gr-1^+^ granulocytes and a low absolute number of F4/80^+^ macrophages while CD137 had the opposite effect (Figure 4E). But the absolute number of granulocytes was not affected when bone marrow cells were treated with CD137 protein in addition to G-CSF (Figure 4E). Similarly, the absolute number of F4/80^+^ macrophages was not significantly reduced when cells were treated with G-CSF in addition to CD137 protein.

### CD137 protein acts on early myeloid progenitor cells

As shown above the CD137 protein-induced increase in the macrophage/granulocyte ratio is at least in part due to its effects on mature bone marrow cells. But CD137 ligand signaling has also been shown to influence proliferation and differentiation of hematopoietic progenitor cells [23]–[25]. Bone marrow cells that were depleted of cells expressing markers of mature cells (lin^−^) and enriched for c-kit, the receptor for stem cell factor, proliferated, formed colonies and differentiated to macrophages [25]. Therefore, the increased macrophage/granulocyte ratio might also be due to influences of the CD137 protein and G-CSF on hematopoietic progenitor cell differentiation.

**Figure 5:**
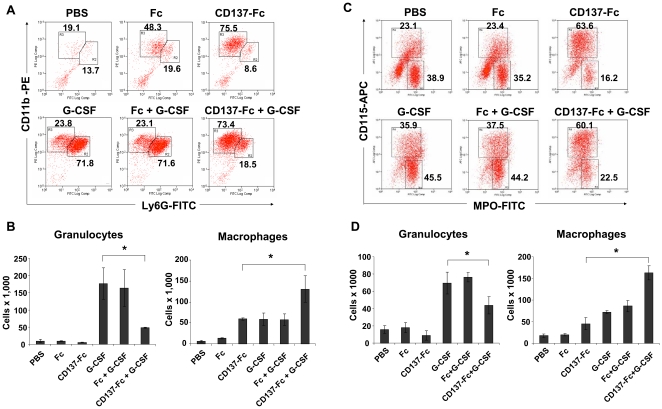
Dominance of CD137 protein-induced monocytic differentiation. 5×10^5^ lin^−^, c-kit^+^ cells at a density of 5×10^5^ cells/ml were cultured on plates that had been coated with 10 µg/ml of Fc or CD137-Fc protein or that were not coated (PBS, medium control). Where indicated, G-CSF was added daily at 10 ng/ml. The percentages (A) and absolute numbers (B) of CD11b^+^, Ly6G^−^ and CD11b^+^, Ly6G^+^ cells, and the percentages (C) and absolute numbers (D) of CD115^+^, MPO^−^ and CD115^−^, MPO^+^ cells were determined by flow cytometry on day 7. (A) and (C) are representatives of five and two experiments, respectively, (B) and (D) are depicted as means ± standard errors of five and two independent experiments, respectively. * p<0.05.

As expected, G-CSF induced granulocytic differentiation in lin^−^, c-kit^+^ cells whereas CD137-Fc protein induced monocytic differentiation. An predominant induction of granulocytic differentiation by G-CSF has been shown before [28], [29]. When both factors, G-CSF and CD137-Fc were added together the same high percentage of monocytic cells (CD11b^+^, Ly-6G^−^) were obtained as with CD137 protein alone. Although the addition of G-CSF to CD137 protein-stimulated cells increased the percentage of granulocytes (CD11b^+^, Ly6G^+^) from 8.6% to 18.5%, it was still much lower than that with G-CSF alone (71.8%), (Figure 5A). This indicated that, in the presence of CD137 protein, G-CSF had less influence on the differentiation of the progenitor cells, while CD137 protein was dominant and more potent in inducing monocytic differentiation.

The dominance of CD137 protein in inducing monocytic differentiation was also evident from the absolute cell numbers since drastically fewer granulocytes were present when CD137 protein was added to G-CSF-treated cells (47,800 vs 176,600), (Figure 5B). However, this was not a mutual two-way competition, because both factors together gave rise to more monocytic cells than CD137 protein alone (130,700 vs 59,400), (Figure 5B). This means that G-CSF contributed to enhanced proliferation and survival of progenitor cells but CD137 protein redirected differentiation from the granulocytic to the monocytic lineage. These results were confirmed by measuring expression of myeloperoxidase (MPO) and CD115 (M-CSF receptor), another set of granulocytic versus monocytic markers, respectively (Figure 5C,D).

**Figure 6:**
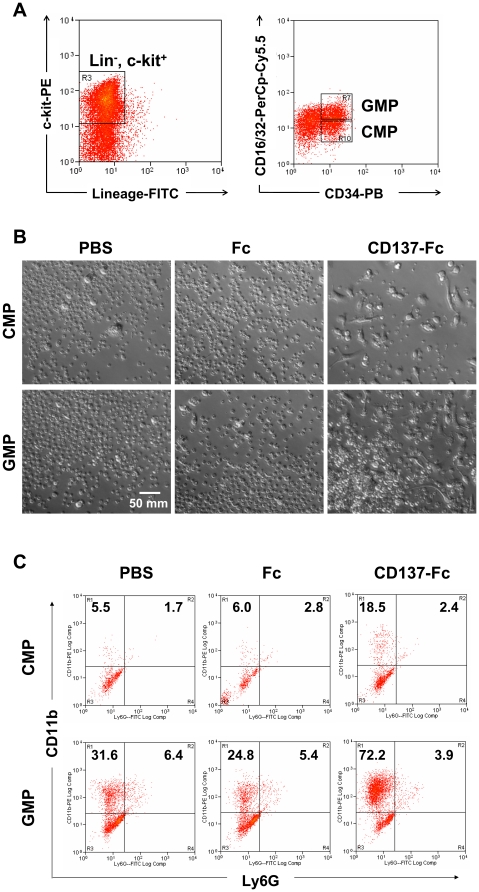
CD137 protein induces monocytic differentiation in early myeloid progenitor cells. (A) Lin^−^, c-kit^+^ cells (boxed area of left panel) were sorted for CD34^+^, CD16/32^high^ (GMP) and CD34^+^, CD16/32^low^ (CMP) cells (right panel). (B) 5×10^4^ CMP or GMP at a density of 2.5×10^5^ cells/ml were cultured for 7 days on plates that had been coated with 10 µg/ml of Fc or CD137-Fc protein or that were not coated (PBS, medium control). Photographs were taken on day 7 at magnification of ×200. Scale bar = 50 µm. Please note that the small round particles are cell debris as evidenced by their small size and the holes in the membranes. (C) The percentages of CD11b^+^, Ly6G^−^ and CD11b^+^, Ly6G^+^ cells of (B) were determined by flow cytometry.

CD137 protein is able to induce monocytic differentiation in even more primitive hematopoietic progenitor cells. Common myeloid progenitors (CMP: lin^−^, c-kit^+^, CD34^+^, CD16/32^low^) and granulocyte macrophage progenitors (GMP: lin^−^, c-kit^+^, CD34^+^, CD16/32^high^) were isolated from lin^−^, c-kit^+^ cells by cell sorting (Figure 6A). When the cells were stimulated with immobilized CD137-Fc protein they showed an increased adherence and a change to macrophage morphology (Figure 6B). Also, the monocytic cell population (CD11b^+^, Ly6G^−^) was increased but not the granulocytic cell population (CD11b^+^, Ly6G^+^), (Figure 6C). These data together with published reports [7], [8], [11], [12] confirm CD137 as a potent monocytic growth and differentiation factor.

## Discussion

CD137 ligand signaling has been shown to regulate multiple activities of myeloid cells. It induces activation of human and murine monocytes and macrophages, monocyte migration, survival and even proliferation. Further, it induces human monocyte to DC differentiation and maturation of immature DCs [8]. Also, the proliferation and differentiation of hematopoietic progenitor cells is regulated by CD137 ligand signals although there are conflicting data on its precise effects on hematopoietic progenitor cells [24], [25].

Given the numerous influences of CD137 ligand signals on monocytes, macrophages and DCs we hypothesized that the biology of granulocytes, another type of myeloid cells, may also be affected. Indeed, that turned out to be the case. However, contrary to monocytic cells, the percentage of granulocytes among bone marrow cells was reduced after treatment with recombinant CD137 protein.

The reduced percentage of granulocytes among total bone marrow cells could be due to three potential mechanisms: (1) induction of cell death in granulocytes, (2) prolongation of monocyte survival, and (3) skewing of differentiation of hematopoietic progenitor cells towards the monocytic lineage. Induction of granulocyte cell death could be ruled out but prolongation of macrophage survival and enhanced differentiation of progenitor cells to macrophages both contributed to the increased number of monocytic cells.

This means that CD137 ligand signaling does not affect granulocytes directly as is the case for hematopoietic progenitor cells and monocytic cells, at least not in the murine system. But the reduced percentage of granulocytes upon treatment with by CD137 protein was a result of the selective growth stimulus for monocytic cells. In line with this finding is that there is no evidence of CD137 ligand expression on murine granulocytes, and that addition of G-CSF, the prototypic granulocyte growth factor, raised the percentage of granulocytes among total bone marrow cells. Adding G-CSF and CD137 protein together resulted in significantly more cells after a 7-day culture period than either factor alone. The fact that the combination almost had an additive effect on the number of live bone marrow cells indicates that each factor may have promoted survival of distinct subpopulations.

As described above the increased number of monocytic cells among total bone marrow cells resulted from an increased survival of mature cells and from an increased monocytic differentiation of progenitor cells. The opposing effects of G-CSF and CD137 protein in reducing or enhancing the macrophage/granulocyte ratio among total bone marrow cells was not observed for hematopoietic progenitor cells indicating that the increased number of monocytic cells among total bone marrow cells resulted mainly from promoting survival of mature granulocytes and macrophages, respectively. Both G-CSF and CD137 protein were able to induce proliferation of hematopoietic progenitor cells and to induce granulocytic and monocytic differentiation, respectively. The combination of G-CSF and CD137 protein generated significantly (p<0.05) more cells during a 7-day culture period than either factor alone but the majority of these cells were of the monocytic lineage based on the CD11b^+^, Ly6G^−^, and the CD115^+^, MPO^−^ phenotype. This means that both factors contributed to an enhanced hematopoietic progenitor cell proliferation but CD137 protein was dominant over G-CSF in inducing monocytic differentiation in the newly emerging cells.

A pathological situation where the hematopoietic activities of CD137 may play a role could be after injuries, especially burns. Thermal injuries often lead to monocytosis that is accompanied by neutropenia, due to a shift of progenitor differentiation away from granulocytic towards monocytic differentiation [30], [31]. The activities of CD137 protein identified in this study would be compatible with such a notion.

G-CSF is being used therapeutically for treatment of neutropenia [32]. The combination of G-CSF and CD137 ligand agonists, especially at varying concentrations may allow a precise fine-tuning of myelopoiesis, depending on whether more granulocytes or more macrophages are required for a specific therapeutic setting.

The above described enhancement of monocytic cell numbers is likely mediated by signaling through CD137 ligand (reverse signaling) on progenitor cells inducing monocytic differentiation. In mature monocytes/macrophages CD137 ligand signaling induces release of M-CSF, the classical monocyte growth factor and thereby greatly extends the survival of these cells [12]. In addition, CD137 ligand signaling induces monocyte growth, proliferation and endomitosis [11], [14]. However, the CD137 receptor/ligand system may also bias myeloid cell composition towards macrophages, via forward signaling. In the human system, CD137 can be expressed on mature granulocytes in certain physiological and/or pathological conditions and signaling through CD137 has been shown to inhibit granulocyte survival [26]. On human eosinophil granulocytes CD137 expression is inducible by an unidentified T cell-derived factor, and crosslinking of CD137 overrides the pro-survival effects of GM-CSF and IL-5 [33]. Similarly, human neutrophil granulocytes express CD137 constitutively, and crosslinking of CD137 overrides the pro-survival effects of GM-CSF and induces apoptosis [34]. However, we could not detect CD137 expression on murine granulocytes (not shown). This could mean that the monocytic/granulocytic cell ratio in the murine system is mainly controlled by the differentiation of progenitor cells.

The CD137 receptor/ligand system has been shown to mediate monocyte proliferation and endomitosis [11], [19]–[21], DC differentiation [17], [18] and maturation [19]–[21]. Also, CD137 ligand signaling has been found to regulate proliferation and differentiation of hematopoietic progenitor cells [23]–[25]. The data of the present study on the interactions of G-CSF and CD137 on total bone marrow cells and bone marrow progenitor cells add to the growing recognition that CD137 and its ligand regulate multiple aspects of hematopoiesis, in particular of myelopoiesis.
